# Editorial: Molecular components of store-operated calcium entry in health and disease, volume II

**DOI:** 10.3389/fncel.2023.1300412

**Published:** 2023-10-09

**Authors:** Francisco Javier Martin-Romero, Agnese Secondo, Joanna Gruszczynska-Biegala

**Affiliations:** ^1^Institute of Molecular Pathology Biomarkers, University of Extremadura, Badajoz, Spain; ^2^Department of Neuroscience, “Federico II” University of Naples, Naples, Italy; ^3^Laboratory of Molecular Biology, Mossakowski Medical Research Institute, Polish Academy of Sciences, Warsaw, Poland

**Keywords:** SOCE, Orai, STIM1, STIM2, TRP channels

The role of Ca^2+^ homeostasis in brain function, as well as its dyshomeostasis in brain dysfunction, has been recognized for a long time. However the involvement of each single ionic protein in modulating a specific pathway remains unknown. This enormously limits the possibility of a proper pharmacological intervention. Of interest, each channel may work alone or in collaboration with other ionic entities to regulate a variety of physiological processes that often involve the molecular components of store-operated calcium entry (SOCE).

Previous studies showed that AQP4 and TRPV4 are essential components of a molecular complex that is critically involved in the hypotonicity-induced rise in [Ca^2+^]i in astrocytes. Sucha et al. demonstrated that the interaction between AQP4 and TRPV4 channels may play an important role in edema formation after ischemia *in vivo* and under ischemia-like conditions such as oxygen and glucose deprivation. In fact, their simultaneous deletion results in a smaller brain lesion at D1 but an equal tissue damage at D7 when compared to the controls. Therefore, the simultaneous deletion of AQP4 and TRPV4 channels appears to be protective only during the acute phase of the insult. This study highlights the importance of maintaining both neuronal and glial ionic homeostasis in preventing damage, thus identifying two new putative pharmacological targets, and their interaction, in brain ischemia.

Patients suffering from neurological diseases, including cerebral ischemia, have difficulty or delay in eliciting the swallowing reflex, which in turn can lead to food particles entering the lungs and aspiration pneumonia. Another role of the TRPV4 channel turned out to be inducing the swallowing reflex (Hossain et al.). This channel is expressed on sensory nerve fibers (SLN; superior laryngeal nerve) and SLN-afferent neurons innervating areas associated with swallowing. In rats, administration of an agonist of TRPV4 increased the number of swallowing reflexes, which were significantly attenuated after prior administration of a TRPV4 antagonist. In their research, the authors postulate that TRPV4 may be a new therapeutic target for oropharyngeal dysphagia.

In another article in this issue, An et al. investigated the synaptic connectivity of TRPV1-positive trigeminal afferents within the lateral parabrachial nucleus (LPBN) of the rat. TRPV1 is a receptor associated with pain and temperature perception. The study focuses on understanding how these afferents, which convey sensory information from the face and mouth, form connections in the LPBN. Using rats and immunological techniques, the researchers traced the pathways of these trigeminal afferents and examined their synaptic connections within the LPBN. The authors found that all TRPV1+ boutons in the LPBN establish simple synaptic contacts with 1–2 postsynaptic dendrites, i.e., without engaging in complex synaptic arrangements, using a completely different strategy than that found in the trigeminal caudal nucleus. Thus, the research could have implications for understanding pain perception, temperature regulation, and other sensory functions related to the face and mouth.

Another channel from the TRP channel family is TRPM, a monovalent sodium-selective TRP channel expressed in many components and involved in the modulation of several functions including sweet, bitter, and umami signaling pathways. Of note, TRPM4 and TRPM5 are also involved in taste-evoked signaling. Using live cell imaging, Banik and Medler showed that the release of calcium from the endoplasmic reticulum in the Ca^2+^-induced Ca^2+^ release (CICR) process activates TRPM4 in broadly responsive cells belonging to type III taste cells. TRPM4 participates in calcium signaling by linking PLCβ3/IP3R1 signaling to the activation of L-type VGCCs, but not P/Q, N, and T-type VGCCs. Subsequently, increased calcium influx through L-type VGCCs activates taste-evoked calcium responses.

The article by Ben Dhaou et al. discusses a study on neural stem cells (NSCs) from the adult mouse area postrema and their capacity for self-renewal through a mechanism involving SOCE, with particular attention to the influence of the hormone leptin. Neural stem cells are essential for brain repair and maintenance, and their self-renewal is crucial for ongoing neurological health. This study explores how calcium signaling, mediated by TRPCs, Orai1, and STIM1, plays a critical role in regulating the self-renewal of these stem cells. The study highlights the impact of leptin, a hormone whose effects on energy homeostasis depends on the area postrema, decreases SOCE and reduces self-renewal of NSCs in this area. Since leptin dysregulation is associated with obesity, the study underscores the importance of SOCs in metabolic disorders.

To summarize, in recent years, attention has been paid to the role of SOC channels in neurons, which include Orai and TRP (transient receptor potential) channels as an effective regulator of Ca^2+^ levels in the ER due to their interaction with STIM proteins (Serwach and Gruszczynska-Biegala, 2020). As a result of neuronal excitation, glutamate is released into the synaptic cleft, resulting in an increased influx of Ca^2+^ into the cytoplasm both from the extracellular space through the VGCC channels and from the ER in the CICR process. A positive signaling loop exists between TRPM4, L-type VGCC, and ryanodine receptors that involves CICR, thereby modulating the taste signal. The TRPC1 and Orai1 channels play a major role in maintaining the activity of NSCs that sustain neurogenesis. Via TRPV1, orofacial nociceptive information is transmitted directly to postsynaptic neurons in the lateral parabrachial nucleus (LPBN). In turn, TRPV4 present in SLN-afferent neurons is involved in triggering the swallowing reflex, and in interaction with AQP4 it may play a detrimental role in the acute phase of brain ischemia through the formation of edema. Thus, the Research Topic “*Molecular components of store-operated calcium entry in health and disease, volume II*” is a resource for researchers and clinicians interested in SOC channels, which in both cellular physiology and pathology are involved in more pathways than just the regulation of calcium signals.

## Author contributions

FM-R: Conceptualization, Funding acquisition, Writing—original draft, Writing—review and editing. AS: Conceptualization, Funding acquisition, Writing—original draft, Writing—review and editing. JG-B: Conceptualization, Funding acquisition, Writing—original draft, Writing—review and editing.
